# Spontaneous solar water splitting with decoupling of light absorption and electrocatalysis using silicon back-buried junction

**DOI:** 10.1038/s41467-020-17660-0

**Published:** 2020-08-06

**Authors:** Hui-Chun Fu, Purushothaman Varadhan, Chun-Ho Lin, Jr-Hau He

**Affiliations:** 1 Computer, Electrical, and Mathematical Sciences and Engineering, King Abdullah University of Science and Technology, (KAUST), Thuwal, 23955-6900 Saudi Arabia; 2 KAUST Solar Center, KAUST, Thuwal, 23955-6900 Saudi Arabia; 3 Department of Materials Science and Engineering, City University of Hong Kong, Kowloon, Hong Kong SAR

**Keywords:** Energy, Solar fuels, Photocatalysis

## Abstract

Converting sunlight into a storable form of energy by spontaneous water splitting is of great interest but the difficulty in simultaneous management of optical, electrical, and catalytic properties has limited the efficiency of photoelectrochemical (PEC) devices. Herein, we implemented a decoupling scheme of light harvesting and electrocatalysis by employing a back-buried junction (BBJ) PEC cell design, which enables >95% front side light-harvesting, whereas the electrochemical reaction in conjunction with carrier separation/transport/collection occurs on the back side of the PEC cell. The resultant silicon BBJ-PEC half-cell produces a current density of 40.51 mA cm^−2^ for hydrogen evolution by minimizing optical, electrical, and catalytic losses (as low as 6.11, 1.76, and 1.67 mA cm^−2^, respectively). Monolithic fabrication also enables three BBJ-PEC cells to be connected in series as a single module, enabling unassisted solar water-splitting with a solar-to-hydrogen conversion efficiency of 15.62% and a hydrogen generation rate of 240 μg cm^−2^ h^−1^.

## Abstract (web summary)

The simultaneous management of optical, electrical, and catalytic properties is challenging for photoelectrochemical devices. Here, authors design Si back-buried junction photoelectrodes that can be series connected for unassisted water-splitting with a high solar-to-hydrogen efficiency of 15.62%.

## Introduction

The simultaneous conversion and storage of abundant, but intermittent solar energy have been envisioned as a sustainable approach for producing clean and renewable fuels. Photoelectrochemical (PEC) water splitting is particularly attractive, as hydrogen is an efficient and clean fuel for electricity generation^1–5^. Unfortunately, challenges such as efficiency and cost have obstructed extensive employment of solar hydrogen/fuel cell hybrid systems^6,7^. Silicon (Si), with its optimal bandgap (*E*_*g*_ = 1.1 eV) for the solar spectrum, is the most investigated and highly used photon absorber for PEC applications^8–14^. However, there are several significant issues in employing Si as a photoelectrode, including the necessity of using light-blocking electrocatalysts to promote the hydrogen- and/or oxygen-evolution reaction (HER, OER)^8^, and its limited bandgap, which cannot straddle both the reduction (0.0 V_RHE_) and oxidation (1.23 V_RHE_) potentials of water^9,10^.

In current state-of-the-art PEC device design, all significant functionalities, including light absorption, surface protection, and electrolysis occur on the same side of the device, whereas the opposite side of the device is only used for electrical connections^3^. The requirement of high mass loading of electrocatalysts to reduce the electrocatalytic overpotential and the necessity of surface protection layers leads to significant parasitic light absorption that jeopardizes the PEC efficiency^8,15–18^. As a result, most PEC devices demonstrated thus far feature a design in which one side is overused while the other is underutilized^19,20^. One promising route to address this issue involves spatially and functionally decoupling the optical absorption and catalytic activity of the photoelectrode. Recently, Vijselaar et al.^15,18^ demonstrated the partial decoupling of light harvesting and electrocatalysis in Si-micro/nanopillars by selective integration of electrocatalysts on the high aspect ratio structures, but such designs are complex and difficult to adopt in conventional PEC devices.

Additionally, unassisted water splitting requires a potential of ~1.7–1.8 V to meet the thermodynamic requirements for water splitting (1.23 V at 20 °C) and other necessary overpotentials to drive the HER and OER reactions^1,21^. However, Si as a single photoelectrode is not capable of producing such high photovoltage^21^. Accordingly, in the literature, most Si-based PEC demonstrations are half-cell based, which only simulates a part of the PEC system (either a photoanode or photocathode) and not the overall solar water-splitting process^13,14,22,23^. Moreover, the half-cell device designs demonstrated thus far cannot be easily connected in series, marking contemporary efforts in spontaneous water splitting still very much proof-of-concept^13,14,22,23^. To work around this constraint, tandem designs have been developed, in which materials with different bandgaps are combined for unassisted solar hydrogen production. However, this strategy is not straightforward due to the lattice and thermal mismatch complications in growing higher bandgap materials on Si^13,20^.

To address these issues, we demonstrate a universal scheme of spatially and functionally decoupling the light harvesting and electrocatalysis components in a PEC system by fabricating a back-buried junction (BBJ) based on single crystalline Si featuring the electrocatalyst and carrier separation/collection junctions on the back side of the device, whereas the front surface is dedicated to light harvesting. The BBJ-PEC cell with an antireflective (AR) layer and micropyramidal surface without electrocatalyst light-blocking can collect over 95% of the solar spectrum, and thus the current density can be maximized to 40.51 mA cm^−2^ with minimal optical, electrical, and catalytic current losses (6.11, 1.76, and 1.67 mA cm^−2^, respectively) for HER, demonstrating the well-optimized PEC water-splitting design. By integrating three BBJ-PEC photoelectrodes in series into a single module, we are also able to achieve unassisted solar water splitting. The resultant device exhibits an open-circuit potential of ~1.83 V and a current density of 12.6 mA cm^−2^, which translates to a solar-to-hydrogen (STH) efficiency of 15.62% for an unassisted Si-based PEC cell. Finally, for further optimization, we provide an in-depth analysis of the various possible loss mechanisms (optical, electrical, and catalytic) for photon-to-hydrogen conversion in the PEC device.

## Results

### Design of the BBJ-PEC cell

Decoupling the optical and catalytic interfaces in PEC water splitting has recently been explored with different approaches^18,24,25^. Partial decoupling of light absorption and electrocatalysis have also been demonstrated by spatially selective deposition of electrocatalysts on the surface of nanostructures with high aspect ratios^18,25^, but such designs are complex in nature and challenging to integrate into conventional PEC devices. To address these issues, we adopted a BBJ device design, which was recently demonstrated to achieve a record solar-to-current conversion efficiency in a photovoltaic (PV) cell^26^. Though the BBJ design has made tremendous progress for PV applications, employing such cells for PEC water splitting is not straightforward.

**Fig. 1:**
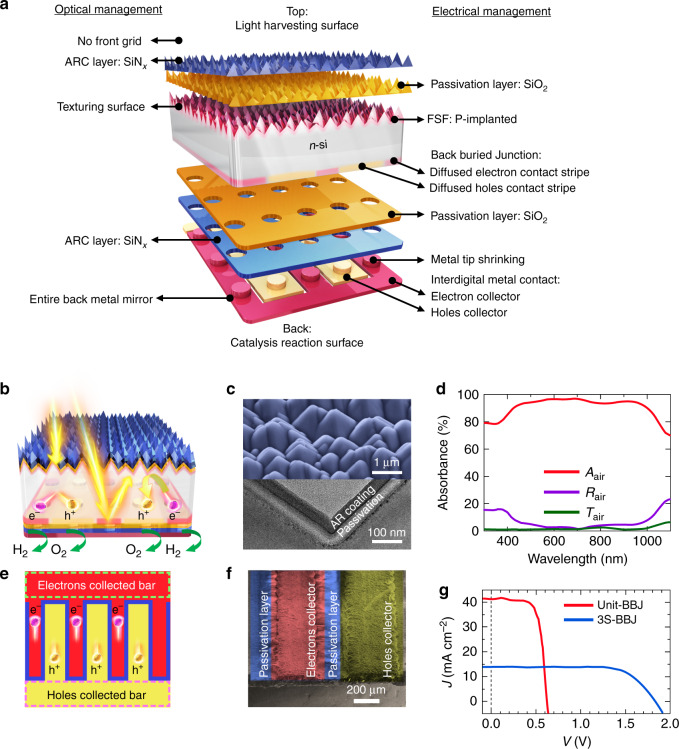
The BBJ–PEC Cell. **a** Schematic of the components of the BBJ cell. **b** Cross-section and the optical trails of the BBJ cell. **c** The top panel displays an SEM image of the front surface of a BBJ cell textured with micropyramidal structures. The bottom panel shows a high-magnification STEM image of the micro-pyramid surface, which features uniform passivation (SiO_2_) and AR coating (SiN_*X*_) layers. **d** The absorbance, reflectance, and transmittance spectra measured from the front pyramid surface. **e** The plane-view schematic of interdigitated dopant pattern of the BBJ cell. **f** A plane-view false color SEM image of the interdigitated metal grids on the back surface of the cell. **g** PV characteristics of a unit- and a 3S-BBJ cell.

Figure 1a displays a schematic diagram of a unit BBJ-PEC cell (see “Methods” for complete fabrication details). Specifically, the front side of the BBJ device is optimized for light harvesting without any catalyst shadowing effect, whereas the back side of the device plays the vital role of electrocatalysis, enabling the comprehensive light-harvesting surface to be exposed on the front side completely (Fig. 1b).

Enhancement of the optical characteristics is realized by texturing the Si surface with micropyramidal structures (top panel of Fig. 1c), as well as applying an AR layer of 60 nm SiN_*x*_ (bottom panel of Fig. 1c) after the deposition of passivation layer to both the front and back surfaces of the cell, ensuring broadband omnidirectional light-harvesting with minimal reflection. The overall contribution of photon management scheme can be observed from the light-harvesting capability of the device, as demonstrated in the surface absorbance spectra (Fig. 1d), measured by ultraviolet–visible spectrophotometry in the air. The corresponding equation is shown below:

1 $$A_{\mathrm{air}}\left( \lambda \right) = 100{\mathrm{\% }} - R_{\mathrm{air}}\left( \lambda \right) - T_{\mathrm{air}}\left( \lambda \right),$$

in which *A*_air_(*λ*), *R*_air_(*λ*), and *T*_air_(*λ*) are the light absorbance, reflectance, and transmittance of the BBJ-PEC cell, respectively. The absorbance spectra of the BBJ-PEC cell demonstrates over 95% absorption of the solar spectrum from 300 to 1100 nm, which has not been realized using a conventional PEC design to date.

Photo-excited carrier recombination is another significant loss factor limiting PEC efficiency, and hence an optimized electrical design is vital for avoiding excessive recombination and improving the minority carrier collection (i.e., holes in the n-type Si used in this study). To achieve this goal, we fabricated the BBJ by doping boron (B) and phosphorous (P) via local ion-implantation in an interdigitated dopant pattern (Fig. 1e) on the back (catalysis) surface of the device to fabricate a p^+^-Si emitter (hole collection bar with ~400 µm wide) and a n^+^-Si back surface field (BSF acts as an electron collection bar with ~200 µm wide) to improve minority carrier collection. In this manner, we were able to produce a cascade-type band structure to enhance the separation/transport efficiency of photo-excited carriers in the photoelectrode^26^. Additionally, a thin layer of implanted P (dopant thickness of 70 nm) was formed as the front-surface field (FSF) (shown in Fig. 1a) of the entire micropyramidal surface (where the recombination velocity is high) to reflect the holes down to the hole collector at the back of the PEC cell, thus reducing the surface recombination. After ion-implantation, we deposited a 40-nm SiO_2_ passivation layer before the AR coating to saturate dangling bonds on both front and back surfaces. Supplementary Fig. 1 shows the cross-sessional images of SiN_*x*_/SiO_2_/Si structure obtained by scanning transmission electron microscopy (STEM) with energy dispersive X-ray spectroscopy/electron energy loss spectroscopy (EDX/EELS) mapping, illustrating the intact passivation and AR layers.

We then deposited the interdigitated metal contacts/catalysts (Pt on n^+^-Si for HER and Ni on p^+^-Si for OER) on the catalysis reaction surface at the back side of the cell, which aligns with the interdigitated dopant pattern of the BBJ to collect the photo-excited electrons and holes, thus facilitating the PEC water splitting. We inserted all the contact grids/catalysts at the back of the PEC device for two reasons. First, it can maximize the light absorption by eliminating shadowing losses caused by the catalyst/metal contacts (i.e., the optical design). Second, it brings the catalysts in close contact with the buried junction located at the catalysis reaction surface of the PEC cell in order to efficiently extract the photo-excited carriers simultaneously for the PEC water splitting (i.e., the electrical/catalyst design). The overall electrical management scheme aims to reduce contact resistance and carrier recombination, thus facilitating HER and OER at the back (catalysis reaction) surface of BBJ-PEC cells. Moreover, inserting uniform interdigitated junction/metal grid contacts (Fig. 1f) on the entire catalysis surface shortens the path of the photo-excited carriers to the contacts by creating more junction areas, allowing the charge carriers to be efficiently separated, transported, and collected by the back grids. Meanwhile, the metal grids (up to 70% coverage) also serve as back mirror for internal photon reflection to promote the long-wavelength absorption by the photoelectrodes, as shown in Fig. 1b.

### Photoelectrochemical characterization

Before characterizing the PEC water-splitting behavior of our design, we measured the PV properties of the BBJ cell under one sun AM 1.5 G illumination in the air (Fig. 1g). The advantages obtained from the BBJ design were evidenced by a short-circuit current density (*J*_SC_) of 40.98 mA cm^−2^, an open-circuit voltage (*V*_OC_) of 0.62 V, a fill-factor (FF) of 74.46%, and thus a PV conversion efficiency (*E*_ff_) of 18.92%. The external quantum efficiency (EQE) and current loss analysis (Supplementary Fig. 2) of the BBJ cell exhibits outstanding optical and electrical performance, with maximum absorption in the AM 1.5 G solar spectrum, as envisioned by integrating the EQE spectral response over the photon flux in the AM 1.5 G solar spectrum. The current loss analysis demonstrates the limited parasitic losses due to the adoption of the decoupled light harvesting and electrocatalysis management scheme and the BBJ device architecture.

In addition to these advantages over the conventional vertical junction PEC cell structure, the BBJ design with a horizontal array of p–n junctions at entire back surface can benefit the PEC cell assembly by allowing the simultaneous interconnection of a series of three BBJ cells (3S-BBJ) to achieve a *V*_OC_ up to 1.83 V (a detailed description of the fabrication process is provided in the “Methods” section), as shown in Fig. 1g (blue curve). Most importantly, the improved *V*_OC_ of 1.83 V by the 3S-BBJ device is higher than the required thermodynamic potential for unassisted photoelectrolysis (~1.23 eV) plus the catalysis overpotential requirements, enabling wireless water splitting^27^.

**Fig. 2:**
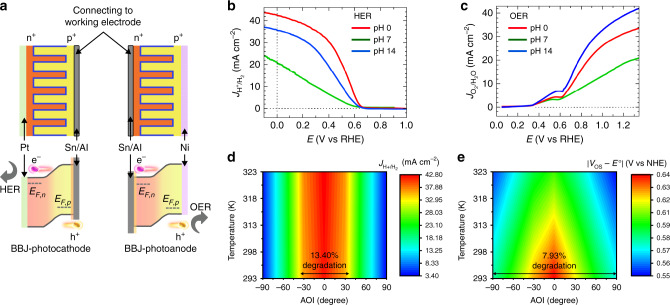
Photoelectrochemical characteristics of a unit BBJ cell. **a** The schematic and band profile of the BBJ–PEC cells. LSV curves under simulated AM 1.5 G illumination in acidic (red line), neutral (green line), and basic (blue line) electrolytes measured by a BBJ unit cell for **b** HER and **c** OER. The AOI and temperature dependent PEC characteristics of **d**$J_{{\mathrm{H}}^ + /{\mathrm{H}}_2}$ and **e** |*V*_OS_ − *E*^0^|. Note that NHE is the normal hydrogen electrode, *V*_OS_ is the onset potential measured at a hydration current of 1 mA cm^−2^, *E*^0^ is the equilibrium water reduction potential, and $J_{{\mathrm{H}}^ + /{\mathrm{H}}_2}$ is the saturation current density at *E*^0^ for HER.

In the BBJ device, both electron and hole collectors were patterned and aligned with respect to the local interdigitated p–n junctions for facilitating water splitting, which successfully improve carrier separation, collection, and electrocatalysis reaction. The local p- and n-junctions are connected to their respective carrier collection bars and centralized at opposite ends of the cell (as shown in Fig. 1e, the electron and hole collection bars are marked by green and purple dashed boxes, respectively). Pt and Ni were deposited by sputtering on at the opposite ends of the cell electrons and holes collection area as the HER and OER electrocatalysts, respectively. Figure 2a shows the schematic and band profile of the BBJ photocathode and photoanode. Supplementary Fig. 3 shows the fabrication schematics of the isolated packing process for BBJ photocathode and photoanode (see “Methods” and Supplementary Fig. 3 for fabrication details), which makes HER and OER work individually from the interdigital BBJs. In order to demonstrate the unassisted water-splitting capability, it is essential to optimize and validate the performance of a unit BBJ-PEC cell for HER and OER half-cell measurements. We analyzed the PEC performance of a unit BBJ photoelectrode using linear sweep voltammetry (LSV) with a standard three-electrode setup in a quartz cell under simulated AM 1.5 G one sun illumination (see “Methods”). The behavior of the photoelectrode was evaluated using standard performance metrics, with potentials calculated relative to the reversible hydrogen electrode (RHE)^28^. It should be noted that the performance of the BBJ-PEC photoelectrode evaluated by this method is only for half-cell metrics and not the overall water-splitting efficiency. We carried out the HER and OER measurements under different pH conditions for a unit BBJ-PEC cell, as it is important for a photoelectrode to perform under a wide range of pH conditions to achieve the balance between the pH requirements of two working electrodes^1,29^. The HER and OER performances in 1 M H_2_SO_4_ (pH ~0), 1 M Na_2_SO_4_ (pH ~7), and 1 M KOH (pH ~14) electrolytes are shown in Fig. 2b, c and summarized in Supplementary Table 1. Under the best performing conditions, a unit BBJ photoelectrode achieved a saturation current density for HER ($J_{{\mathrm{H}}^ + /{\mathrm{H}}_{2}}$) of 40.51 mA cm^−2^ and for OER ($J_{{\mathrm{O}}_2/{\mathrm{H}}_2{\mathrm{O}}}$) of 28.68 mA cm^−2^ at the equilibrium water reduction and oxidation potential, respectively. It should be noted that the non-ideal feature in the onset region of the OER is due to the Ni oxidation reaction (more details on the non-ideal feature of Ni oxidation reaction can be found in the Supplementary Information and Supplementary Fig. 4). In the wide range pH conditions for HER and OER, the BBJ photoelectrode demonstrates the excellent performance, outperforming reported Si-based PEC cell for HER thus far (Supplementary Table 2).

The temperature and angle-of-incidence (AOI) of sunlight on a PEC cell will change through the course of a day and season. Hence it is crucial to analyze temperature- and AOI-dependent PEC performances for real-world deployment. In general, the energy bandgap (*E*_*g*_) of the semiconductor decreases with temperature, which is determined by the Green’s law (more details on the temperature dependence of *E*_*g*_ and the corresponding factor can be found in the Supplementary Information and Supplementary Fig. 5)^30–32^, resulting in the variation of PEC performance under fluctuations of temperature. Similarly, the AOI significantly affects surface geometry-related reflection/scattering. To understand how these variables may affect the PEC performance, we measured the PEC performance of BBJ photocathode at various temperatures (293–323 K) under AM 1.5 G illumination and varying the AOI from 90° to −90° (relative to a normal vector of the light-harvesting plane). The $J_{{\mathrm{H}}^ + /{\mathrm{H}}_2}$ and |*V*_OS_ − *E*^0^| (where the *V*_OS_ is the onset potential measured at the current density of 1 mA cm^−2^, *E*^0^ is the equilibrium water reduction potential) results are shown in Fig. 2d, e and summarized in the Supplementary Table 3.

The increasing temperature induces bandgap shrinkage in semiconductors and promotes more thermally excited carriers^33^. Therefore the semiconductor responds to extra absorption in longer wavelength regions, leading to a slightly increased $J_{{\mathrm{H}}^ + /{\mathrm{H}}_2}$ with temperature. Meanwhile, the increase in temperature can cause the reduction of *V*_OC_ and |*V*_OS_ − *E*^0^| (more detailed discussion is provided in the Supplementary Information). Supplementary Fig. 6 shows the change in the PEC performances as a function of temperature and the corresponding results are summarized in Supplementary Table 4. On the other hand, maximum light absorption should be warranted to maximize the PEC performance. As discussed earlier, the light-harvesting characteristics were optimized and as a result, a broad range of light acceptance was demonstrated with the degradation of <14% in $J_{{\mathrm{H}}^ + /{\mathrm{H}}_2}$ over an AOI of 60° (−30° to 30°). Furthermore, over a wide AOI of 180° (−90° to 90°), |*V*_OS _− *E*^0^| degradation is <8%, which has not previously been achieved. The broad range AOI of the BBJ photoelectrode is due to the dedicated decoupling scheme with pyramidal surface and AR layer.

### Comprehensive analysis of current losses

To gain more insight into the spatial and functional decoupling design of the photoelectrode’s light harvesting and electrocatalysis capabilities, we analyzed the overall solar-to-hydrogen conversion from end-to-end photon-to-fuel pathways and the associated losses by employing theoretical current loss analysis of a unit BBJ-PEC device for HER at pH 0.

**Fig. 3:**
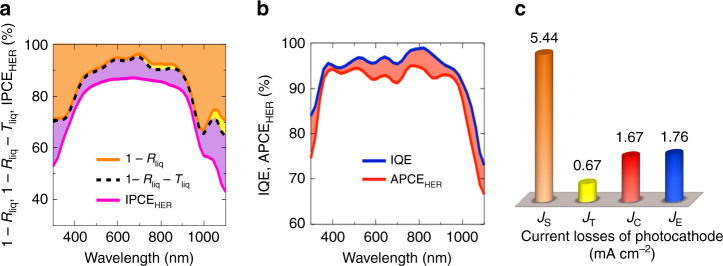
Comprehensive analysis of the current losses by a unit BBJ photocathode for HER at pH 0. **a** Surface absorptance/reflectance/transmittance spectra and IPCE for HER. For the sake of clarity, the loss sources of *J*_S_ and *J*_T_ are shown by the area colored with orange and yellow, respectively. *J*_Int_ is depicted with the color purple. **b** The IQE and APCE_HER_ spectra, in which *J*_C_ is depicted with the color red. **c** Summarized current losses obtained from **a** and **b**. The *J*_Opt_ = *J*_S_ + *J*_T_, and the *J*_Int_ = *J*_E _+ *J*_C_.

There are three fundamental steps of the PEC water splitting for the current loss analysis. First, photo-excitation generates the charge carriers. Second, photo-excited charge carriers are separated and transport to the catalysis reaction surfaces. Third, the charge carriers drive catalytic water reduction or oxidation at the surfaces. In the comprehensive analysis, the current losses can be grouped into the optical current loss (*J*_Opt_) from the first step, and the internal current loss (*J*_Int_) from the last two steps. Furthermore, the *J*_Opt_ is separated into two components: surface reflection loss (*J*_S_) and transmission loss (*J*_T_). For the sake of clarity, the loss sources of *J*_S_ and *J*_T_ are depicted in Fig. 3a by the area colored with orange and yellow, respectively. The corresponding formulas are expressed as below:

2 $$J_{\mathrm{S}} = \frac{q}{{hc}}\int_{300}^{1100} \lambda \cdot {\Phi}(\lambda ) \cdot R_{\mathrm{liq}}\left( \lambda \right) \cdot {\mathrm{d}}\lambda,$$

3 $$J_{\mathrm{T}} = \frac{q}{{hc}}\int_{700}^{1100} \lambda \cdot {\Phi}(\lambda ) \cdot T_{\mathrm{liq}}\left( \lambda \right) \cdot {\mathrm{d}}\lambda,$$

in which *q* is the charge of an electron, *h* is Planck’s constant, *c* is the speed of light in vacuum, *λ* is the photon wavelength, Φ(*λ*) is the AM 1.5 G solar spectrum (300 to 1100 nm), and *R*_liq_(*λ*) and *T*_liq_(*λ*) are the reflectance and transmittance of the BBJ photoelectrode measured in liquid electrolyte, respectively (as shown in Supplementary Fig. 7). The *J*_S_ is due to back scattered photons at the surface of the PEC cell and in the electrolyte, which was calculated to be 5.44 mA cm^−2^ according to Eq. (2). As compared to the literature, the minimal *J*_S_ loss that occurred herein can be attributed to the unique optical and catalytic interface decoupling scheme employed, enabling the ideal optimization of the light harvesting component, which cannot be achieved using the conventional PEC photoelectrode design. With Eq. (3), we calculated the *J*_T_ by optical transmission through the body and the interdigitated electrode gaps of the BBJ device to be as low as 0.67 mA cm^−2^, which can be attributed to the long-wavelength photons (700–1100 nm) with higher transmission than the short wavelength region. The low *J*_T_ value indicates the reflective electrode/catalyst at the back of the photoelectrode (up to 70% coverage) can effectively serve as a mirror to reflect unabsorbed light back to the device.

To further analyze the *J*_Int_, we carried out light absorbance of a unit BBJ cell measured in liquid electrolyte (*A*_liq_(*λ*)) and the incident photon-to-current efficiency (IPCE) of the half-cell, which can be written as:

4 $$A_{{\mathrm{liq}}}\left( \lambda \right) = \left( {1 - R_{{\mathrm{liq}}}\left( \lambda \right) - T_{{\mathrm{liq}}}\left( \lambda \right)} \right) \times 100{\mathrm{\% }}.$$

5 $$\begin{array}{*{20}{l}} {{\mathrm{IPCE}}\left( \lambda \right)} \hfill & = \hfill & {\frac{{{\mathrm{Number}}\,{\mathrm{of}}\,{\mathrm{collected}}\,{\mathrm{carriers}}}}{{{\mathrm{Number}}\,{\mathrm{of}}\,{\mathrm{incident}}\,{\mathrm{photons}}\,{\mathrm{shining}}\,{\mathrm{on}}\,{\mathrm{the}}\,{\mathrm{cells}}}}} \hfill \\ {} \hfill & = \hfill & {\frac{{1240 \times J_{{\mathrm{H}}^ + /{\mathrm{H}}_2}\left( {{\mathrm{mA}}{/}{\mathrm{cm}}^2} \right)}}{{\lambda \left( {{\mathrm{nm}}} \right) \times P_{\mathrm{in}}\left( {{\mathrm{mW}}{/}{\mathrm{cm}}^2} \right)}} \times 100\% } \hfill \end{array}.$$

The IPCE describes the maximum possible efficiency with which incoming photons can produce hydrogen from water, with an implicit assumption that all electrons are used for the HER (and holes for the OER instead of other byproducts or corrosion, assuming that the Faradaic efficiency is 100%). Therefore, the *J*_Int_ can be calculated by the difference between *A*_liq_(*λ*) and IPCE(*λ*) as shown below:

6 $$J_{{\mathrm{Int}}} = J_{\mathrm{E}} + J_{\mathrm{C}} = \frac{q}{{hc}}\int_{300}^{1100} \lambda \cdot {\Phi}\left( \lambda \right) \cdot \left( {A_{{\mathrm{liq}}}\left( \lambda \right) - {\mathrm{IPCE}}\left( \lambda \right)} \right) \cdot {\mathrm{d}}\lambda.$$

It should be noted that the *J*_Int_ can be distributed into two parts: electrical loss (*J*_E_) and catalytic loss (*J*_C_), which can be understood by the absorbed photon’s failure to generate photon-excited carriers and the photon-excited carriers recombining before the catalysis reaction, resulting in *J*_E_, and photon-excited carriers accommodating at the catalyst–electrolyte interface but failing to participate in the surface catalysis reaction, resulting in *J*_C_. Based on Eq. (6), we calculated *J*_Int_ to be 3.43 mA cm^−2^.

To decouple *J*_E_ and *J*_C_ in order to provide an overall dynamic picture of the photo-excited carriers in the bulk of the photoelectrode and in the catalyst/electrolyte interface, we compared the internal quantum efficiency (IQE, measured in air without electrolyte to understand the inherent EQE without considering the catalytic capability in the electrolyte) and the absorbed photon-to-current efficiency (APCE), as shown in Fig. 3b. The IQE is defined as the number of photo-generated carriers contributing to the photocurrent divided by the number of absorbed photons in the materials measured in air, while the APCE is defined by the ratio of the number of photo-generated carriers contributing to the $J_{{\mathrm{H}}^ + /{\mathrm{H}}_2}$for HER to the number of absorbed photons by the BBJ half-cell measured in the electrolyte:

7 $$\begin{array}{*{20}{l}} {{\mathrm{QE}}\left( \lambda \right)} \hfill & = \hfill & {\frac{{{\mathrm{Number}}\,{\mathrm{of}}\,{\mathrm{collected}}\,{\mathrm{photo}} {\hbox{-}} {\mathrm{generated}}\,{\mathrm{carriers}}\,{\mathrm{for}}\,{\mathrm{dry}}\,{\mathrm{cell}}}}{{{\mathrm{Number}}\,{\mathrm{of}}\,{\mathrm{absorbed}}\,{\mathrm{photons}}}}} \hfill \\ {} \hfill & = \hfill & {\frac{{{\mathrm{EQE}}\left( \lambda \right)}}{{A_{{\mathrm{air}}}\left( \lambda \right)}} \times 100\% } \hfill \end{array}.$$

8 $$\begin{array}{*{20}{l}} {{\mathrm{APCE}}\left( \lambda \right)} \hfill & = \hfill & {\frac{{{\mathrm{Number}}\,{\mathrm{of}}\,{\mathrm{collected}}\,{\mathrm{photo}} {\hbox{-}} {\mathrm{generated}}\,{\mathrm{carriers}}\,{\mathrm{for}}\,{\mathrm{electrolysis}}}}{{{\mathrm{Number}}\,{\mathrm{of}}\,{\mathrm{absorbed}}\,{\mathrm{photons}}}}} \hfill \\ {} \hfill & = \hfill & {\frac{{{\mathrm{IPCE}}\left( \lambda \right)}}{{A_{{\mathrm{liq}}}\left( \lambda \right)}} \times 100 \% } \hfill \end{array}.$$

The IQE determines the efficiency of charge separation and transport to the solid–liquid interface. The change in APCE (related to the efficiency of charge separation and transport to the solid–liquid interface and the efficiency of the catalysis reaction at the solid/electrolyte interface) could point to dynamic behavior of photo-excited carriers in both the bulk of general PEC cell and the catalyst–electrolyte interface, since bulk recombination and inactive catalytic reaction can consume the photo-generated carriers. Therefore, the difference in IQE and APCE can account for the *J*_C_ arising from the imbalanced kinetics of the catalysis reaction, in which an electron or a hole transfers and accommodates at the catalyst–electrolyte interface but fails to participate in the surface catalysis reaction. Thus, *J*_C_ can be obtained by integrating Eq. (9).

9 $$J_{\mathrm{C}} = \frac{q}{{hc}}\int_{300}^{1100} \lambda \cdot {\Phi}\left( \lambda \right) \cdot \left( {{\mathrm{IQE}}\left( \lambda \right) - {\mathrm{APCE}}\left( \lambda \right)} \right) \cdot {\mathrm{d}}\lambda.$$

For the sake of clarity, the loss sources of *J*_C_ are depicted in Fig. 3b by the red colored region and calculated to be 1.67 mA cm^−2^. According to Eqs. (6) and (9), a *J*_E_ of 1.76 mA cm^−2^ can calculated by the difference between *J*_Int_ and *J*_C_.

The result of current loss analysis is summarized in Fig. 3c. The optimized optical and electrical designs with minimal current losses achieved herein can be attributed to the spatial decoupling of light absorption and catalytic activity, and the excellent electrical design of the BBJ-PEC cell. Using the current loss analysis, we can physically monitor the dynamics of photo-excited carriers in the bulk of the photoelectrode and at the catalyst/electrolyte interface. It is worth noting that this comprehensive analysis of the current losses is based on the general semiconductor half-cell, which is extendable to any semiconductor photoelectrode.

### Unassisted solar water splitting

**Fig. 4:**
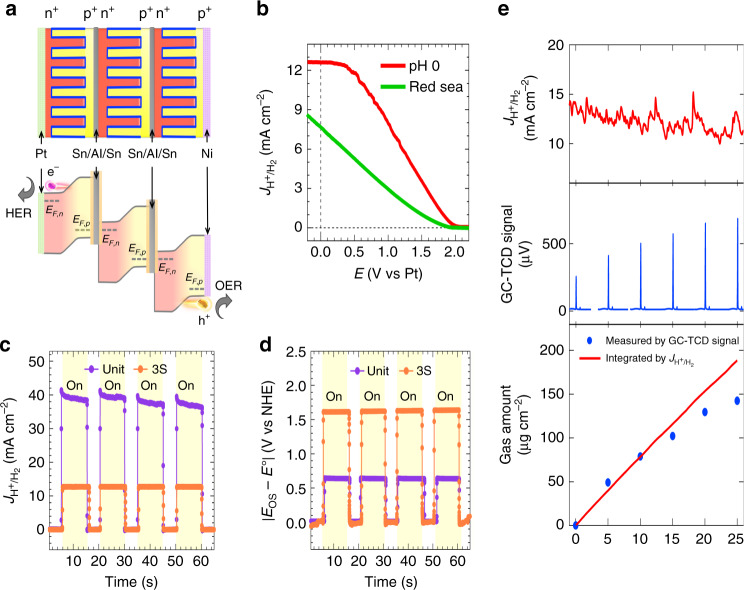
The characterization of the 3S-BBJ–PEC cell. **a** The schematic and band profile of the 3S-BBJ–PEC cell. **b***J*–*E* characteristics of the 3S-BBJ cell in 1 M H_2_SO_4_ (red) and Red Sea water (green) electrolytes. **c** Time-resolved saturated current density and **d** Time-resolved onset potential of the unit- and 3S-BBJ–PEC cells. **e** GC measurement of a 3S-BBJ–PEC cell under AM 1.5 G illumination without bias, where the top panel is the *J*–*T* curve during the GC measurement, the middle panel is the GC with thermal conductivity detector (GC-TCD) signal of hydrogen, and the bottom panel is the amount of hydrogen evolved as a function of time as calculated by integrating the current by the time (red line), and by the H_2_ concentration obtained from the GC-TCD signal (blue dots). PEC performances were measured under simulated AM 1.5 G illumination using a Ag/AgCl reference electrode in 1 M H_2_SO_4_ electrolyte.

The potential requirement to drive wireless water electrolysis is ~1.7–1.8 V^34^. To implement wireless water-splitting, we fabricated monolithic devices by integrating three BBJ-PEC unit cells in series via tin soldering, in which p^+^-Si and n^+^-Si were bonded together (p^+^nn^+^-p^+^nn^+^-p^+^nn^+^) with the Al foil. The advantage of BBJ-PEC cells is that the junction can make series connection laterally on the same surface to remove the shadowing effect. Hence, the maximized light harvesting was obtained after series the connection, which cannot be achieved by conventional vertical junction devices. The schematic and the corresponding band profile for the fully integrated 3S-BBJ-PEC cell is represented in Fig. 4a (see “Methods” for fabrication details; the photograph and fabrication schematic of the isolated packing process for 3S-BBJ devices can be found in Supplementary Fig. 8). The *J*–*V* characteristic of the 3S-BBJ cell measured in air demonstrates a *V*_OC_ of 1.83 V (Fig. 1g), which is well-above the voltage requirement for unassisted water-splitting with the given electrocatalysts.

Finally, we employed a 3S-BBJ-PEC cell in a photocathode configuration for unassisted water splitting in a two-electrode measurement setup (see “Methods”) by connecting the 3S-BBJ-PEC cell to the dark anode (Pt). The PEC reaction was carried out under 1 M H_2_SO_4_ with pH ~0. Bubble formation was visible immediately after illuminating the device under one sun AM 1.5 G simulator, demonstrating spontaneous water splitting without the application of an external bias. Figure 4b and Supplementary Table 5 show the performance of a wireless 3S-BBJ-PEC cell for the HER in 1 M H_2_SO_4_ (red line), which shows the unassisted current density of 12.60 mA cm^−2^. We also demonstrate the time-resolved saturated $J_{{\mathrm{H}}^ + /{\mathrm{H}}_2}$ and |*V*_OS_ − *E*^0^| with chopped illumination in Fig. 4c, d, showing a stable STH conversion over four cycles. As expected, the results show that the 3S-BBJ cell has ~3 times the |*V*_OS_ −  *E*^0^| and ~1/3 the $J_{{\mathrm{H}}^ + /{\mathrm{H}}_2}$ compared to a BBJ unit cell configuration without a series connection. The STH efficiency for the 3S-BBJ photoelectrode without applying an external bias (*V*_app _= 0 V) can be calculated using Eq. (10):

10 $${\mathrm{STH}}(\%) = \frac{{J_{{\mathrm{H}}^ + /{\mathrm{H}}_2} \times (1.23 - V_{{\mathrm{app}}})}}{{P_{{\mathrm{in}}}}},$$

in which *P*_in_ is the incident optical power shining on the cell. With the unassisted $J_{{\mathrm{H}}^ + /{\mathrm{H}}_2}$ of 12.60 mA cm^−2^, an STH efficiency of 15.62% was achieved, which is the highest efficiency ever reported for Si-based unassisted PEC water splitting. In order to simulate real-world solar hydrogen production, we employed water obtained from the Red Sea as an electrolyte (~pH 8.1), and in outdoor midday (12:00 p.m.) illumination conditions to drive a series connected 3S-BBJ cell, yielding an STH efficiency of 11.33% (green line in Fig. 4b and Supplementary Table 5).

### Unassisted water-splitting stability

In general, it is well known that technologically matured semiconductors such as Si-based photoelectrodes undergo severe corrosion/oxidation during the water-splitting process^33–35^. Despite the demonstration of high half-cell efficiencies and improved stabilities under various electrolytic conditions, the PEC stability during the overall water-splitting is still vital and challenging. In literature, the stability issues has been partially addressed by depositing compact and continuous metal oxide overlayers such as TiO_2_ using atomic layer deposition (ALD)^34,35^. The deposition of protective metal-oxides has been shown to be successful under nominal current densities to extend the device lifetime, and hence we adopted the surface protection of ALD-TiO_2_ for unassisted water-splitting. To improve the PEC stability, we carried out the ALD deposition of TiO_*x*_ (~140 nm) surface protection layers directly on the BBJ-contact pads before the sputter deposition of Pt (5 nm) for HER purpose. More details on the ALD-TiO_*x*_ is provided in the “Methods” section. The resultant 3S-BBJ-PEC cell under unassisted water-splitting conditions demonstrates 40 h of stability (Supplementary Fig. 9), then the current density decreases due to the corrosion.

Gases evolved from the reactor during the stability measurements were collected with an airtight syringe and analyzed immediately using gas chromatography (GC) to confirm the as-claimed efficiencies (see “Methods”). The top panel of Fig. 4e shows the current density-time ($J_{{\mathrm{H}}^ + /{\mathrm{H}}_2}$ − *T*) curve measured during the gas analysis and the middle panel of Fig. 4e displays the thermal conductivity detector (TCD) signal from the GC measurements. The quantity of generated H_2_ as a function of time can be calculated by two different ways: One is integrating the $J_{{\mathrm{H}}^ + /{\mathrm{H}}_2}$ by the time (red line in the bottom panel of Fig. 4e) and the other is calculating the signals from TCD (blue dots in the bottom panel of Fig. 4e, and the details are shown in Supplementary Fig. 10). These two calculated results demonstrate consistent increase in H_2_ amount in the reactor headspace during the overall water-splitting reactions, which shows that for 20 min of continued operation, the gas evolution density was ~80 μg cm^−2^ under one sun illumination. More importantly, the quantity of gases obtained from the GC measurements matches well with the quantity of integrated electrons-to-H_2_ carriers, with the Faradaic efficiency of >90% for the initial period of 15 min.

Since the photoelectrode was corroded after operation for 40 h, further transmission electron microscopy (TEM) analysis was carried out to understand the failure mechanism on the surface protection of TiO_*x*_/Pt compact layer. The TEM result shows (Supplementary Fig. 11) the photocorrosion and etching of TiO_*x*_/Pt layer that leaded to the cracks in the compact ALD layers and failed to protect the underlying surface. The severe corrosion of 3S-BBJ-PEC could be due to the high current density (~280–300 mA) at the electrodes/electrolytes interface (~1 cm^2^). It should be noted that the total current from the 3S-BBJ-PEC should pass through the limited contact/electrocatalyst in this configuration as shown in Fig. 1a. To address the issue, we employed Pt-sputtered (5 nm)/Ti foil (0.01 mm) on the electron collection bar (see “Methods” for fabrication details) before immersing the 3S-BBJ-PEC cell in the electrolyte (1 M H_2_SO_4_). This idea is similar to the recent surface protection strategy demonstrated by Varadhan et al.^36^, in which a thin Ni-foil (0.125 mm) directly attached to the electrolysis surface acts as both the OER catalyst and the surface protection layer to extend the device lifetime. Herein with Pt/Ti treatment, the resultant 3S-BBJ-PEC cell under two-electrode configuration with 1 M H_2_SO_4_ as the electrolyte exhibits the initial unassisted current density ($J_{{\mathrm{H}}^ + /{\mathrm{H}}_2}$) of 10.5 mA cm^−2^ with the STH efficiency of ~13% (in Supplementary Fig. 12). More importantly, the 3S-BBJ photocathode becomes stabilized with <10% drop in $J_{{\mathrm{H}}^ + /{\mathrm{H}}_2}$ over the period of 100 h, demonstrating highly efficient and stable PEC water-splitting reaction.

## Discussion

In summary, spatial decoupling of light absorption and catalytic activity using the BBJ design allows independent optimization of the light absorption, carrier separation/transport, and PEC catalytic reaction, leading to light absorption of >95% of the solar spectrum and a current density of 40.51 mA cm^−2^ in PEC half-cells for HER—all of which is made possible by achieving minimal optical, electrical, and catalytic current losses (6.11, 1.76, and 1.67 mA cm^−2^, respectively). An additional advantage provided by the BBJ design is its ability to be constructed into a monolithic PEC device based on an interconnected series of the cells, achieving a *V*_OC_ of up to 1.83 V high enough for unassisted solar water-splitting. Thus, the resultant HER under one sun illumination exhibits an STH efficiency of 15.62%. Furthermore, real-world Red Sea water splitting in outdoor midday illumination conditions produced a current density of 9.14 mA cm^−2^ for HER, with an STH efficiency of 11.33%. The efficient BBJ-PEC cell described herein demonstrates promising performance, taking us a step closer to real-world solar-to-hydrogen production.

## Methods

### BBJ cell fabrication

The BBJ cell (~12 × 2 cm^2^) is constructed from n-type crystalline silicon (c-Si) wafer with a thickness of 165 ± 40 μm. We began by texturing the front surface with micropyramidal structures via wet etching to facilitate omnidirectional broadband light absorption and scattering^35^. To reduce recombination on the surface, the micropyramids were lightly P-doped to build an FSF layer. For charge carrier collection, both electron and hole collection occur at the back side of the BBJ cell. B and P dopants were locally ion-implanted to produce p^+^-Si emitter and n^+^-Si BSF with 400 and 250 μm in width, respectively, at the back side of the cell along the interdigitated dopant pattern. The gap between the p^+^- and n^+^-Si contact fingers were connected in parallel with a fixed value of 200 µm. Then, 40-nm-thick SiO_2_ was thermally grown on both the front (micropyramidal) and back (polished) surfaces as a passivation layer to reduce surface recombination losses. Then 60-nm-thick SiN_*x*_ was deposited on both the front and back surfaces by plasma enhanced chemical vapor deposition, which improves the surface reflection and enhances the internal reflectance of the device. Subsequent high-temperature annealing at 950 °C for 5 min was used to activate both types of dopants at the front and back sides of the cell in one step. Then, using a combination of screen-printing and wet-chemical etching, patterned Al electrodes contacted to the p^+^-Si and n^+^-Si were fabricated with a thickness of 2 μm and aligned with the interdigitated dopant pattern.

### 3S-BBJ cell fabrication

For the unit BBJ-PEC cell, electrocatalysts (Pt for HER side and Ni for OER side) were selectively deposited by sputtering on the opposite ends of electron and hole collection bars for making photocathode and photoanode, respectively. Next, the other pole without catalyst deposition (the hole and electron collection bars for photocathode and photoanode, respectively) was connected with the Al foil via tin soldering and then connected to working electrode. After that, the devices were annealed for 1 min at 150 °C under N_2_ ambient. Then the PEC devices were sandwiched between two quartz sheets and sealed using waterproof epoxy (Hysol 11 C), except the electron- and hole-collecting bars (catalyst areas) remaining exposed to the electrolyte for PEC half-cell reactions. A lead is taken out from the epoxy covered macroscopic contact and connected to the counter electrode for the other half-reaction.

A series-based version of the device was produced by connecting a 3-BBJ-PEC cell via tin soldering, in which p^+^-Si and n^+^-Si were bonded together (p^+^nn^+^-p^+^nn^+^-p^+^nn^+^). For the 3S-BBJ photocathode (photoanode) fabrication, the electrocatalysts Pt (Ni) were applied by sputtering on electron (hole) collection bar of the 1st (3rd) of 3S-BBJ cell, and the other pole (hole (electron) collection bar of the 3rd (1st) cell) was connected to Al foil via tin soldering, which will connect to working electrode. On the other hand, for the 3S-BBJ full-cell fabrication, the electrocatalysts Pt and Ni were applied by sputtering on electron collection bar of the 1st cell (photocathode) and hole collection bar of the 3rd cell (photoanode), respectively. We employed Ti foil (0.1 mm) for further protection by applying the Ga-In eutectic as the Ohmic contact with the electron collection bar before sputtering 5-nm-Pt. Then, the devices were annealed at 150 °C for 1 min under N_2_ ambient. Finally, the samples were sandwiched between two quartz sheets and sealed with waterproof epoxy (Hysol 11 C), except the catalyst areas, which remain exposed to the electrolyte. The epoxy was dried (at 80 °C for 30 min) before the measurement. The ALD-TiO_*x*_ films were deposited at 120 °C by using titanium tetraisopropyl oxide (TIIP) as a precursor and H_2_O as the reactant. The dosing time was kept at 1 s pulse for TIIP and 0.1 s for H_2_O. Total number of cycles were fixed at 3000 cyc and thickness of TiO_2_ layer was ~120 nm.

### EDX/EELS mapping in STEM

The samples for scanning transmission electron microscopy (STEM) were prepared by focused ion beam slicing to lift-out the cross-sectional lamella from the micropyramidal surface of the BBJ cell. The high-magnification STEM images were recorded using a FEI Titan 80–300 kV (ST) system operating at 300 kV. The cross-sectional STEM image of the micropyramidal surface is shown in Supplementary Fig. 1a. The EDX/EELS mapping was conducted, in which the Si signal was collected by EDX and the O and N signals were collected by EDX/EELS maps as shown in Supplementary Fig 1b–e.

### Photoelectrochemical LSV

Standard two- and three-electrode setups for wireless and half-cell measurements were employed using a potentiostat (Metrohm Autolab) to measure the LSV of the device. The PEC characteristic of the BBJ photoelectrodes was measured in 1 M H_2_SO_4_ (for HER) and 1 M Na_2_SO_4_ (for OER). For the two-electrode setup, Pt coil and the BBJ photoelectrode were used as the counter and working electrodes, respectively. For the three-electrode setup, Ag/AgCl, Pt coil, and BBJ photoelectrode were used as the reference, counter, and working electrodes, respectively. We used a 150 W halogen-lamp-based solar simulator using an AM 1.5 G filter for illumination. The solar simulator (Newport Corporation, AAA Class) was calibrated before every measurement and, the standard PEC measurement procedure was followed. The distance between each electrode was fixed at 1 cm during measurements to minimize any uncompensated solution resistance loss. All the reported potentials were converted relative to RHE unless specified.

### IPCE measurements

The spot area was 2 × 2 cm. The PEC cells were measured under monochromatic light from 300 to 1100 nm at a chopping frequency of 230 Hz. The IPCE was determined at an applied potential of 0 V vs. NHE.

### GC measurements

The 3S-BBJ photoelectrode was coated with 5 nm Pt on the n^+^ interdigitated area as a catalyst and measured using the standard two-electrode setup under chronoamperometric conditions. The gases evolved were sampled with an Agilent 7890B GC system using an airtight syringe every 5 min to detect the H_2_ concentration. The H_2_ amount was calculated by integrating the continuous $J_{{\mathrm{H}}^ + /{\mathrm{H}}_2}$ output over 25 min from the results of the chronoamperometry measurement, in which two moles of electrons lead to one mole of H_2_.

## Supplementary information

Supplementary Information

## Data Availability

The data that support the plots within this paper and other finding of this study are available from the corresponding author upon reasonable request.
