# Supplementary material for: Spontaneous solar water splitting with decoupling of light absorption and electrocatalysis using silicon back-buried junction
Source: Nat Commun. 2020 Aug 6;11:3930. doi: 10.1038/s41467-020-17660-0 (PMC7411053; doi:10.1038/s41467-020-17660-0)
Supplement: Supplementary file 1 — Supplementary Information [file 41467_2020_17660_MOESM1_ESM.pdf]

Supplementary Information

Spontaneous solar water splitting with decoupling of light absorption and electrocatalysis using silicon back-buried junction

Hui-Chun Fu,[1,2,†] Purushothaman Varadhan,[1,2,†] Chun-Ho Lin,[1] and Jr-Hau He[1,2,3,*]

[1]*Computer, Electrical, and Mathematical Sciences and Engineering, King Abdullah University of Science and Technology, (KAUST), Thuwal 23955-6900 Saudi Arabia*

[2]*KAUST Solar Center, KAUST, Thuwal 23955-6900 Saudi Arabia*

[3]*Department of Materials Science and Engineering, City University of Hong Kong, Kowloon, Hong Kong SAR.*

[†]These authors contributed equally.

[*]Corresponding author: jrhauhe@cityu.edu.hk

Supplementary Figures

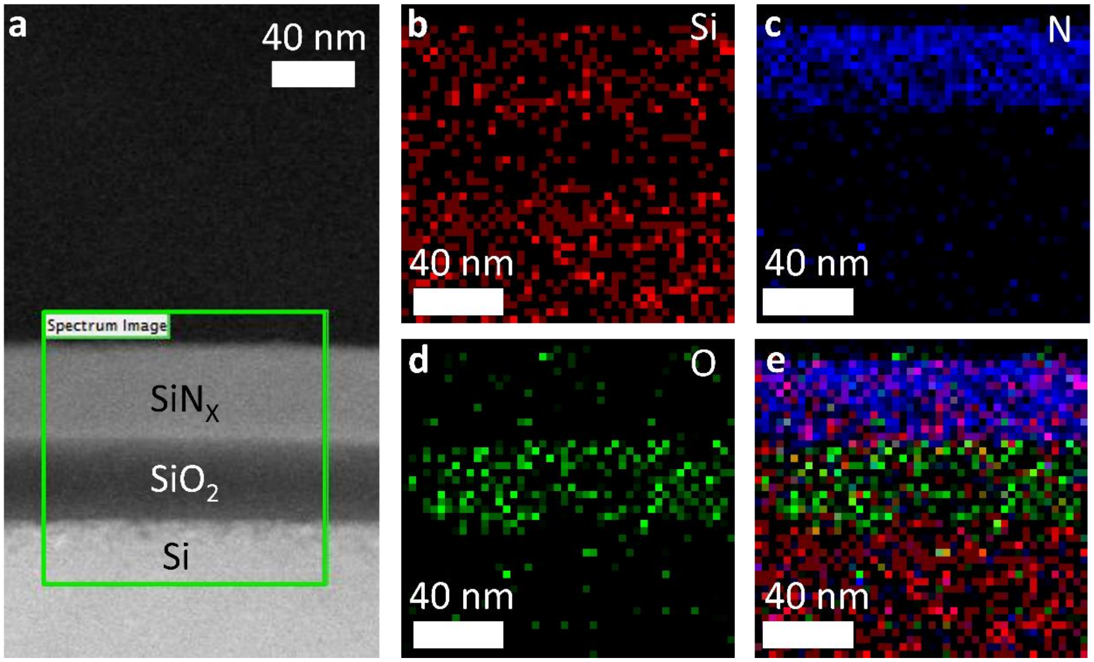

Supplementary Figure 1. STEM analysis of the front surface of the BBJ cell. a STEM image of the **SiN_x/SiO_2/Si** interface and **b-d** the corresponding Si, N, and O elemental concentration mapping. **e** A combined elemental mapping featuring Si in red, N in blue, and O in green.

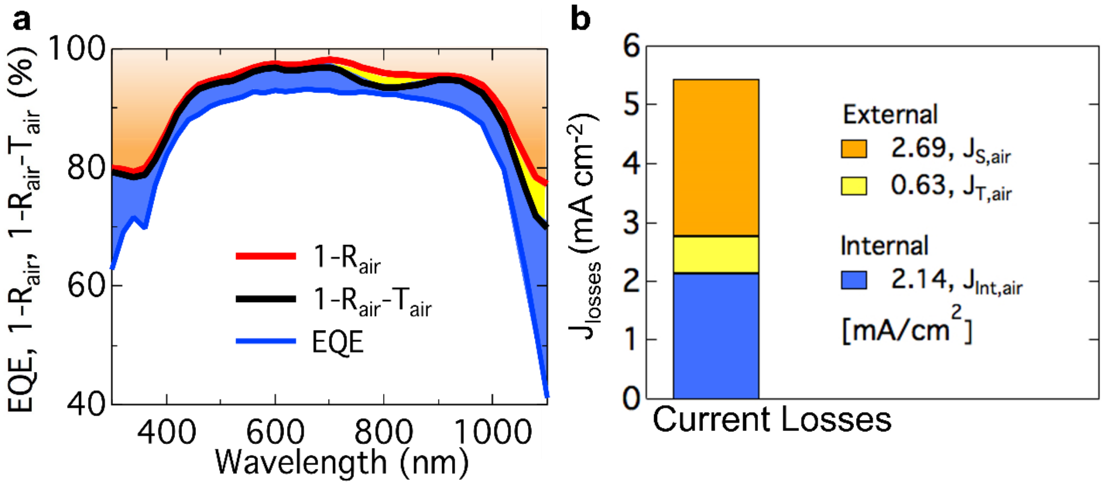

Supplementary Figure 2. Current loss analysis of the PEC cell measured in the air. a $(1 - R_{air})$, $(1 - R_{air} - T_{air})$, and EQE spectra of the BBJ cell. **b** Illustrative example of the current loss sources in a unit BBJ cell. In b the external loss is the total optical loss ($J_{Ext,air} = J_{S,air} + J_{T,air}$), where $J_{S,air}$ is surface reflection loss and $J_{T,air}$ is transmission loss, while the internal (electrical) loss, $J_{Int,air}$, is depicted with the area colored with blue.

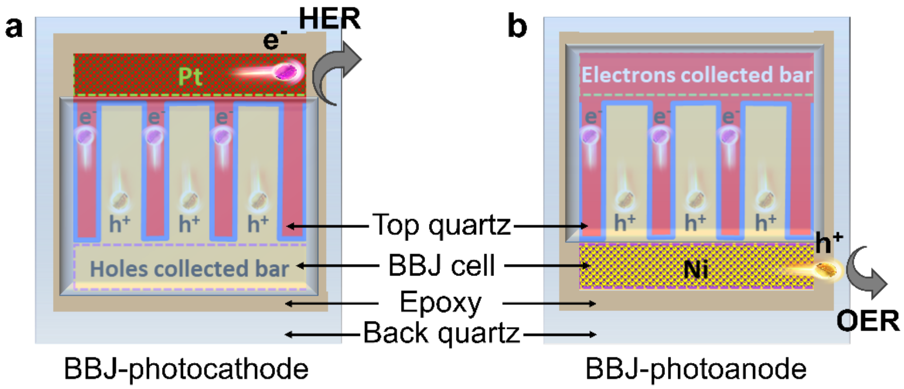

Supplementary Figure 3. The local interdigitated p-n junctions, which the electron collected (n^+-Si with green area) and hole collected (p^+-Si with purple area) bars are centralized at the opposite ends of cell. **a** and **b** are fabrication schematics of BBJ photocathode and photoanode.

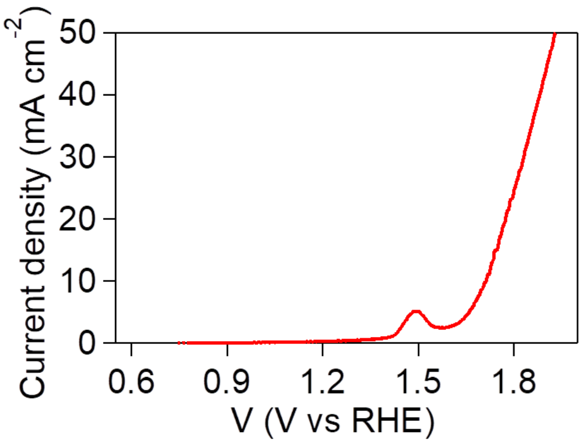

Supplementary Figure 4. The non-ideal feature of Ni oxidation reaction. LSV curve of Sputtered-Ni (50 nm) catalyst measured in 0.5 M KOH. The non-ideal feature at 1.0 V vs. RHE, is due to the oxidation of metallic Ni.

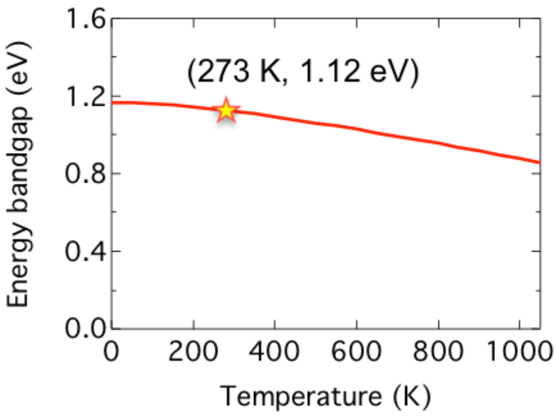

Supplementary Figure 5. Temperature dependence of the energy bandgap of silicon.

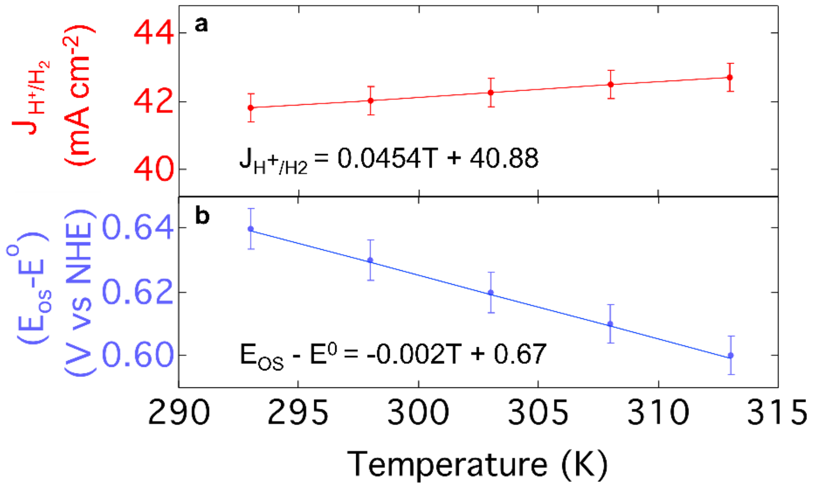

Supplementary Figure 6. PEC performances as a function of temperature at AOI = 0º. a J_{H^+/H_2} (in red) and **b** $|V_{OC} - E^o|$ (in blue).

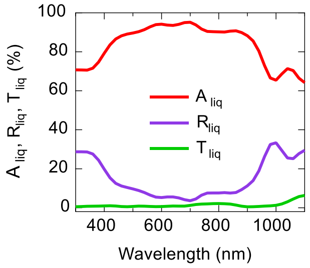

Supplementary Figure 7. Optical characteristics of the BBJ photoelectrode measured in electrolyte (1M H_2SO_4). $A_{liq}(\lambda)$, $R_{liq}(\lambda)$ and $T_{liq}(\lambda)$ are the light absorbance, reflectance, and transmittance measured from the front micropyramidal surface of the BBJ PEC cell in liquid electrolytes, respectively.

a
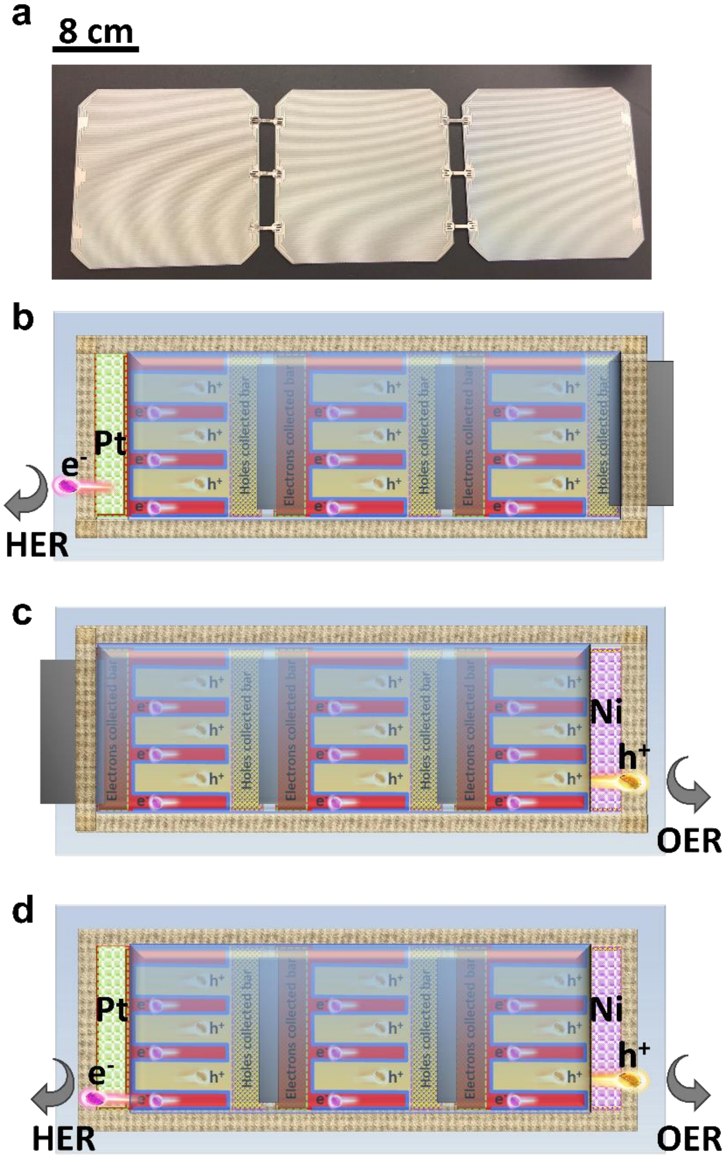

Supplementary Figure 8. The schematic of 3S-BBJ PEC devices for **a** photographic, **b** photocathode and **c** photoanode of half-cell. **d** full-cell 3S-BBJ PEC device.

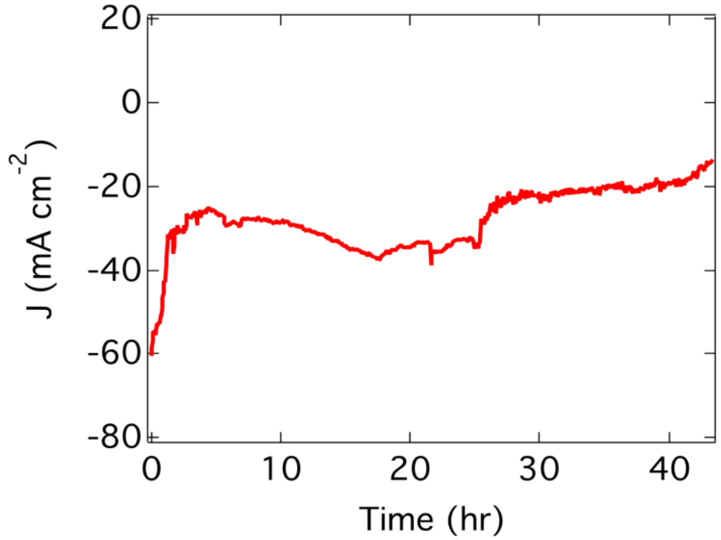

Supplementary Figure 9. A 40-hour-stable HER measured in 1M H_2SO_4 by 3S-BBJ-photocathode with the surface protection of ALD-TiOx.

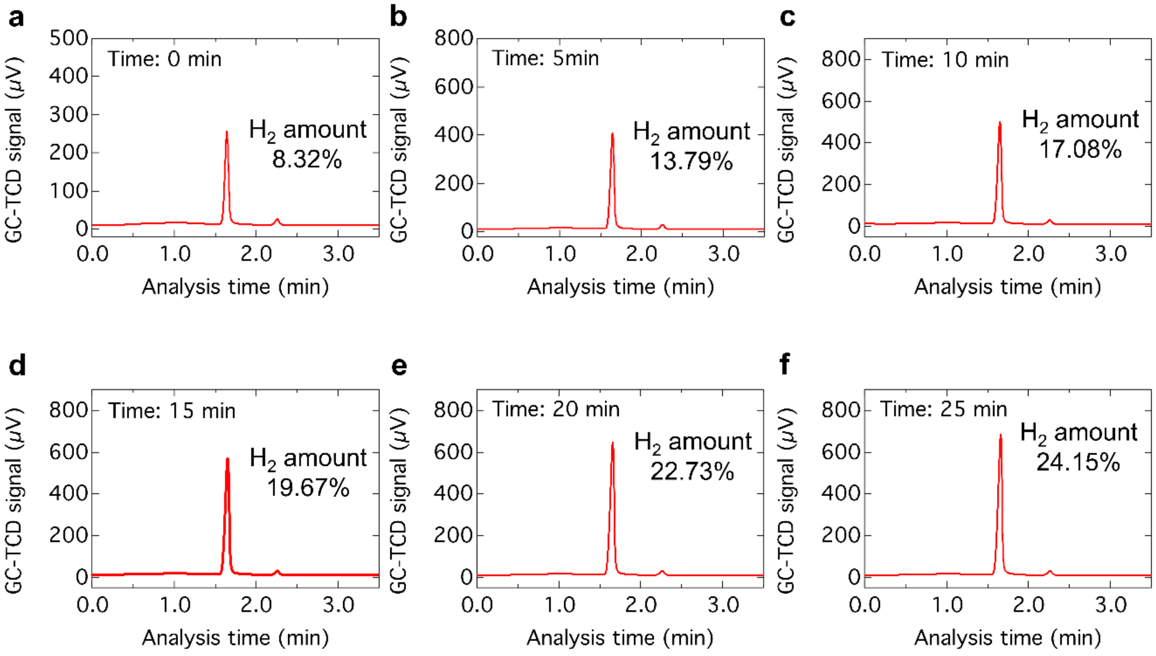

Supplementary Figure 10. Gas chromatography with thermal conductivity detector (GC-TCD) signals of hydrogen with time: GC-TCD signal of HER using a 3S-BBJ photocathode under one sun illumination in 1M H_2SO_4, measured using a two-electrode setup. The GC-TCD signal collected at **a** 0 min, **b** 5 min, **c** 10 min, **d** 15 min, **e** 20 min, and **f** 25 min. Note that the volume of the reactor headspace is 7.3 ml.

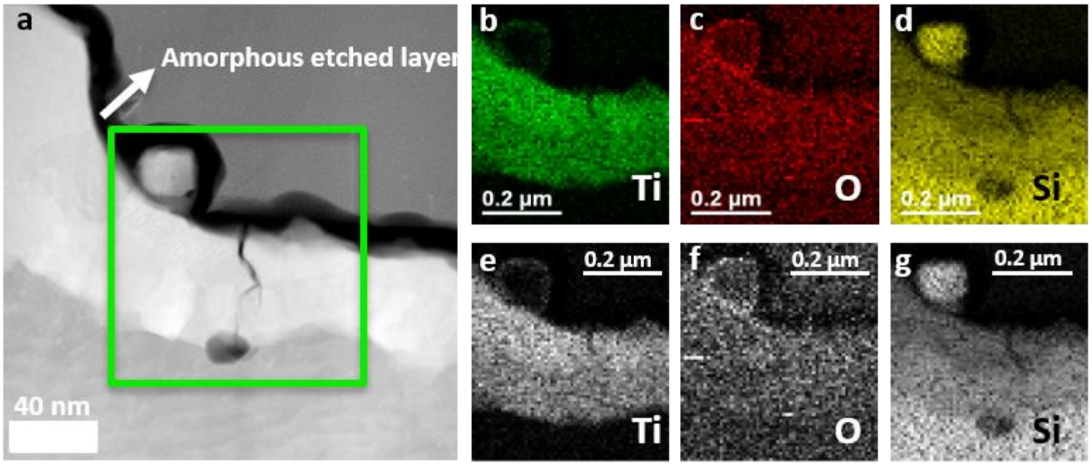

Supplementary Figure 11. **a S**TEM profile of 3S-BBJ-photocathode/electrolyte interface after a 40-hour-stable HER measured in 1M H_2SO_4. **b-d** the corresponding Ti, O, and Si elemental concentration mapping (false-color). **e-g** corresponding Ti, O and Si mapping.

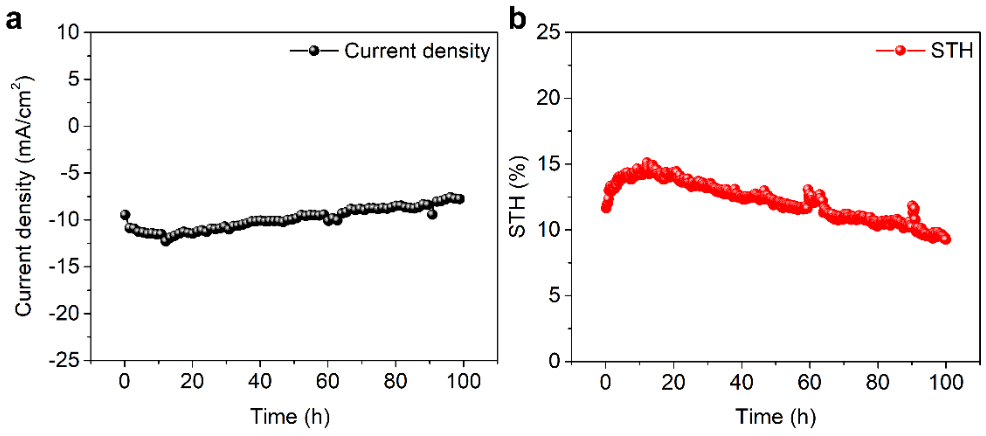

Supplementary Figure 12. A 100 hr stability measurements has been carried out with 3S-BBJ photocathode with a thin Ni-foil (0.125 mm) directly attached to the electrolysis surface acts both as the OER catalyst as well as the surface protection layer under 0.5M H_2SO_4 at one-sun illumination without apply extra bias.

Supplementary Tables

Supplementary Table 1. Hydrogen and oxygen evolution characteristics of a unit BBJ photoelectrodes in acidic, neutral, and basic electrolytes under AM 1.5G illumination. The onset potential was measured at 1 mA cm^{-2}.

BBJ-PEC cells	*Electrolytes*	J_{H^+/H_2}<br>J_{O_2/H_2O}<br>(mA cm^{-2})	$\lvert V_{OS} - E^0 \rvert$<br>$\lvert V'_{OS} - E^{0'} \rvert$<br>(V)
Cathode	pH 0	40.51	0.62
	pH 7	21.19	0.60
	pH14	35.28	0.61
Anode	pH 0	20.25	0.25
	pH 7	8.57	0.24
	pH14	28.68	0.29

Supplementary Table 2. The best reported J_{H^+/H_2} using Si-based PEC photocathodes with the vertical junction.[1-8]

Si-based PEC cells	Catalyst	Electrolyte	pH	J_{H^+/H_2} (mA cm^{-2})	Ref.
ppn$^+$-Si	Pt	0.5M H_2SO_4	0	36.7	1
a-Si/c-Si	Pt	1M H_2SO_4	0	33.49	2
n-i-p Si	Pt	0.5 M KHP	4	11.6	8
pn$^+$-Si	Pt	1M KOH	14	22	3
pn$^+$-Si	Pt	1 M $HClO_4$	0	22	5
a-Si:H/a-Si:H/mc-Si:H	Pt	0.1 M and 1 M KOH	14	7.7	4
n$^+$np$^+$-Si	Pt	1 M $HClO_4$	0	30.9	7
n$^+$p-Si	Pt	1 M $HClO_4$	0	34.8	6

Supplementary Table 3. Hydrogen evolution parameters of the BBJ-PEC cell at different AOI and temperatures under one sun intensity.

AOI (degree)	Temperature (K)	J_{H^+/H_2} (mA cm^{-2})	$\lvert V_{OS} - E^0 \rvert$ (V vs NHE)
	293	3.61	0.58
	298	3.63	0.57
-90	303	3.65	0.56
	308	3.67	0.56
	313	3.69	0.55
	293	18.09	0.60
	298	18.19	0.59
-60	303	18.29	0.58
	308	18.39	0.57
	313	18.49	0.57
	293	36.19	0.62
	298	36.39	0.61
-30	303	36.58	0.60
	308	36.78	0.59
	313	36.98	0.58
	293	41.79	0.63
	298	42.02	0.62
0	303	42.25	0.62

	308	42.47	0.61
	313	42.70	0.60
	293	35.74	0.61
	298	35.94	0.60
30	303	36.13	0.60
	308	36.33	0.59
	313	36.52	0.58
	293	17.87	0.59
	298	17.97	0.58
60	303	18.06	0.57
	308	18.16	0.57
	313	18.26	0.56
	293	3.57	0.57
	298	3.59	0.56
90	303	3.61	0.56
	308	3.63	0.55
	313	3.65	0.54

Supplementary Table 4. Rate of change of performance parameters as a function of temperature for the BBJ-PEC cell.

Unit	$dJ_{H^+/H_2}/dT$ (mA cm^{-2}K^{-1})	$d\|Vos\text{-}E^0\|/dT$ (mV K^{-1})
BBJ-PEC cell	0.045	-2.0

Supplementary Table 5. Wireless hydrogen and oxygen evolution characteristics by the 3S-BBJ cell under AM 1.5G illumination. The onset potential was measured at 1 mA cm^{-2}.

3S-BBJ cell	Electrolytes	J_{H^+/H_2} (mA cm^{-2})	$\lvert V_{OS} - E^0 \rvert$ (V vs NHE)	STH (%)
Cathodes	pH 0	12.60	1.83	15.62
	Red Sea	9.14	1.83	11.33

Supplementary Discussion

EQE Characterization and current loss analysis: To provide a complete picture of the the BBJ cell, we analyzed the external quantum efficiency (EQE), reflectance ($R_{air}(\lambda)$), and absorbance ($A_{air}(\lambda) = [1 - R_{air}(\lambda) - T_{air}(\lambda)] \times 100\%$]) spectra of the BBJ cell measured in air, as shown in Supplementary Fig. 2a. The EQE is defined as the power ratio between the collected photo-generated carriers for dry cells to the incident optical power density shining on the cell:

$$EQE(\lambda)\% = \frac{Collected\ photo - generated\ carriers\ for\ dry\ cell}{Incident\ photons\ shinning\ on\ the\ cells}$$

$$= \frac{1240 \times J(mA/cm^2)}{\lambda\ (nm) \times P_{in}(mW/cm^2)} \times 100\% \qquad (1)$$

The measurement using different excitation sources enables the investigation of semiconductor materials in the energy range spanning infrared (IR), visible, and ultraviolet (UV) light (λ =300–1100 nm).

The current losses of the BBJ cell were classified into external ($J_{Ext,air}$) and internal losses ($J_{Int,air}$). The main external loss is due to optical losses, which are distributed into two different parts. The first part is the surface reflection loss ($J_{S,air}$), which was calculated to be 2.69 mA cm^{-2} from Supplementary Equation 2 due to the light scattering and reflection from the top (light-harvesting) surface of the BBJ cell. The second part is the transmission loss ($J_{T,air}$) of 0.63 mA cm^{-2} through the body of the photoelectrode and the gaps between the interdigitated electrodes at the bottom of the BBJ cell, particularly as the transmission loss is severe in the long wavelength region (700–1100 nm), which was calculated by employing Supplementary Equation 3. The $J_{S,air}$ and $J_{T,air}$ are depicted in Supplementary Fig. 2a in the area colored with orange and yellow, respectively. Hence, the total optical loss ($J_{Ext,air} = J_{S,air} + J_{T,air}$) was calculated to be 3.32 mA cm^{-2}, as shown in Supplementary Fig. 2b.

$$J_{S,air} = \frac{q}{hc}\int_{300}^{1100} \lambda \cdot \Phi(\lambda) \cdot R_{air}(\lambda) \cdot 100\% \cdot d\lambda \qquad (2)$$

$$J_{T,air} = \frac{q}{hc}\int_{300}^{1100} \lambda \cdot \Phi(\lambda) \cdot T_{air}(\lambda) \cdot 100\% \cdot d\lambda \qquad (3)$$

The internal loss component can be obtained by the difference between $A_{air}(\lambda)$ and $EQE(\lambda)$, according to Supplementary Equation 4, which can be understood by the

absorbed photons' failure to generate photo-excited carriers and/or the recombination of the photo-excited carriers before being collecting by the out circuit.

$$J_{Int,air} = \frac{q}{hc}\int_{\lambda_1}^{\lambda_2} \lambda \cdot \Phi(\lambda) \cdot (A_{air}(\lambda) - EQE(\lambda)) \cdot 100\% \cdot d\lambda \qquad (4)$$

Our analysis of J_{Int} is depicted in Supplementary Fig. 2a with the area colored with blue.

The non-ideal feature in the onset region is due to the Ni oxidation reaction. The non-ideal feature in the onset region is due to the Ni oxidation reaction. In the case of photoanode, we use Ni as the electrocatalyst, which in turn oxidizes near to the turn-on potential of the BBJ-PEC cell. Such non-ideal feature has been seen in many literatures where Ni or NiOx as the electrocatalyst are used.[9] In addition, we have cross-checked the same result by sputter deposition of Ni on n^{++}-Si substrate. The cyclic voltammetry (CV) of the sample measured under 0.5 M of KOH shows similar feature which is due to the oxidation reaction of Ni.

Temperature dependence of the energy bandgap ($\boldsymbol{E_g}$). Generally, the E_g for most semiconductors decrease as the temperature increase.[10,11] This behavior can be attributed to the increasing interatomic spacing when the amplitude of the atomic vibrations increases due to the increased thermal energy. The decreasing potential seen by the electrons in the material in turn reduces E_g. Therefore, the E_g can be determined with Green's formula, and the expression for J_{sc} and V_{oc} can be written in terms of temperature (T) as below:[11]

$$E_g(T) = E_g(0) - \frac{\alpha T^2}{T+\beta} \qquad (5)$$

$$V_{OC} = \frac{kT}{q} ln\left[\frac{J_{SC}}{J_0} + 1\right] \qquad (6)$$

$$J_0 = AT^3 exp^{(-\frac{E_g}{kT})} \qquad (7)$$

in which $E_g(0) = 1.166$ eV, $\alpha = 4.73 \times 10^{-4}$ eV K-1, $\beta = 636$ K, J_0 is the reverse saturation current density, *k* is the Boltzmann constant, and *A* is a temperature independent constant. According to Supplementary Equation 5, the temperature dependence of the energy bandgap for silicon can be obtained from the electrical behavior of the PEC cell under a thermal gradient, as shown in Supplementary Fig. 5.

Temperature-dependent PEC performance. We demonstrated the PEC performances of J_{H^+/H_2} and $|V_{OS} - E^0|$ as a function of varied temperature from 293–323 K with an AOI

of 0° in Supplementary Fig. 6. According to the linear fitting of the experimental results, the rate of change in J_{H^+/H_2} and $|V_{OS} - E^0|$ with temperature (*i.e.*, $dJ_{H^+/H_2}/dT$ and $d|V_{OS} - E^0|/dT$) were 0.045 mA cm^{-2} K and -2.0 mV/K as shown in Supplementary Table 4, which is consistent with Green's formula. Therefore, it is necessary to increase the overpotential of the HER at high temperatures. The consistency of the electrical characteristics of the semiconductor and PEC cell can be obtained in real-world temperature variation, compared with crystalline Si-based cell which has been demonstrated.[11,12]

Stability measurement of unit BBJ-photocathode. We have carried out the extended chronoamperometric (CA) stability test for a single unit BBJ-PEC cell in a photocathode configuration at 0.5 M H_2SO_4 electrolyte (with Pt foil at photoanode). The result shows that the BBJ photocathode is stable over 40 hours with the current up to 22 mA cm^{-2} at 0 V vs RHE. More robust design for improving the stability is under investigation. The stability results are incorporated in the revised version of the manuscript and also given below. As shown in Supplementary Fig. 9.

Supplementary References

1 Ding, Q. *et al.* Designing efficient solar-driven hydrogen evolution photocathodes using semitransparent MoQ_xCl_y (Q = S, Se) catalysts on Si micropyramids. *Adv. Mater.* **27**, 6511-6518 (2015).

2 Wang, H. P. *et al.* High-performance a-Si/c-Si heterojunction photoelectrodes for photoelectrochemical oxygen and hydrogen evolution. *Nano Lett.* **15**, 2817-2824, (2015).

3 Bae, D. *et al.* Protection of Si photocathode using TiO_2 deposited by high power impulse magnetron sputtering for H_2 evolution in alkaline media. *Sol. Energ. Mat. Sol.C* **144**, 758-765 (2016).

4 Urbain, F. *et al.* Multi junction Si photocathodes with tunable photovoltages from 2.0 V to 2.8 V for light induced water splitting. *Energy Environ. Sci.* **9**, 145-154 (2016).

5 Seger, B. *et al.* Using TiO_2 as a conductive protective layer for photocathodic H_2 evolution. *J. Am. Chem. Soc.* **135**, 1057-1064 (2013).

6 Fan, R., Dong, W., Fang, L., Zheng, F. & Shen, M. More than 10% efficiency and one-week stability of Si photocathodes for water splitting by manipulating the loading of the Pt catalyst and TiO2 protective layer. *J. Mater. Chem. A* **5**, 18744-18751 (2017).

7 Yin, Z., Fan, R., Huang, G. & Shen, M. 11.5% efficiency of TiO_2 protected and Pt catalyzed n^+np^+-Si photocathodes for photoelectrochemical water splitting: manipulating the Pt distribution and Pt/Si contact. *Chem. Comm.* **54**, 543-546 (2018).

8 Lin, Y. *et al.* Amorphous Si thin film based photocathodes with high photovoltage for efficient hydrogen production. *Nano Lett.* **13**, 5615-5618 (2013).

9 Sun, K. *et al.* A Stabilized, Intrinsically Safe, 10% Efficient, Solar-Driven Water-Splitting Cell Incorporating Earth-Abundant Electrocatalysts with Steady-State pH Gradients and Product Separation Enabled by a Bipolar Membrane. *Adv. Energy Mater.* **6**, 1600379 (2016).

10 Chen, Z., Dinh, H. & Miller, E. *Photoelectrochemical water splitting standards, experimental methods, and protocols* (Springer-Verlag, New York, 2013).

11 Singh, P. & Ravindra, N. M. Temperature dependence of solar cell performance-an analysis. *Sol. Energ. Mat. Sol. C* **101**, 36-45 (2012).

12 Bensalem, S., Chegaar, M. & Aillerie, M. Solar cells electrical behavior under thermal gradient. *Energy Procedia* **36,** 1249-1254 (2013).
